# Exploring the Intestinal Microbiota and Metabolome Profiles Associated With Feed Efficiency in Pacific Abalone (*Haliotis discus hannai*)

**DOI:** 10.3389/fmicb.2022.852460

**Published:** 2022-03-17

**Authors:** Wenchao Yu, Yisha Lu, Yawei Shen, Junyu Liu, Shihai Gong, Feng Yu, Zekun Huang, Weiguang Zou, Mingcan Zhou, Xuan Luo, Weiwei You, Caihuan Ke

**Affiliations:** ^1^State Key Laboratory of Marine Environmental Science, College of Ocean and Earth Sciences, Xiamen University, Xiamen, China; ^2^Fujian Key Laboratory of Genetics and Breeding of Marine Organisms, Xiamen University, Xiamen, China

**Keywords:** *Haliotis discus hannai*, feed efficiency, intestinal microbiota, metagenome, enzyme activity

## Abstract

Feed efficiency (FE) is critical to the economic and environmental benefits of aquaculture. Both the intestines and intestinal microbiota play a key role in energy acquisition and influence FE. In the current research, intestinal microbiota, metabolome, and key digestive enzyme activities were compared between abalones with high [Residual feed intake (RFI) = −0.029] and low FE (RFI = 0.022). The FE of group A were significantly higher than these of group B. There were significant differences in intestinal microbiota structures between high- and low-FE groups, while higher microbiota diversity was observed in the high-FE group. Differences in FE were also strongly correlated to variations in intestinal digestive enzyme activity that may be caused by *Pseudoalteromonas* and *Cobetia*. In addition, *Saprospira*, *Rhodanobacteraceae*, *Llumatobacteraceae*, and *Gaiellales* may potentially be utilized as biomarkers to distinguish high- from low-FE abalones. Significantly different microorganisms (*uncultured beta proteobacterium*, BD1_7_clade, and *Lautropia*) were found to be highly correlated to significantly different metabolites [DL-methionine sulfoxide Arg-Gln, L-pyroglutamic acid, dopamine, tyramine, phosphatidyl cholines (PC) (16:0/16:0), and indoleacetic acid] in the high- and low-FE groups, and intestinal trypsin activity also significantly differed between the two groups. We propose that interactions occur among intestinal microbiota, intestinal metabolites, and enzyme activity, which improve abalone FE by enhancing amino acid metabolism, immune response, and signal transduction pathways. The present study not only elucidates mechanisms of variations in abalone FE, but it also provides important basic knowledge for improving abalone FE by modulating intestinal microbiota.

## Introduction

Feed efficiency (FE) is an essential trait in aquacultural animals because feed accounts for 30–70% of production costs in the aquaculture industry (De Verdal et al., 2017). Improved FE of animals will not only reduce feeding costs and increase profitability but also reduce the environmental impact of large-scale farming (Huntington and Hasan, 2009). Residual feed intake (RFI) reflects the difference between the actual feed intake of the animal and the expected feed intake for growth and maintenance of body weight. Therefore, an animal with a low RFI that consumes less feed per unit of body weight than expected is efficient, while an animal with a high RFI that consumes more feed than expected is inefficient (Patience et al., 2015). RFI was first proposed in 1963 as an index for evaluating the animal FE in beef cattle (Koch et al., 1963) and has subsequently been used to assess FE in other species (Dai et al., 2017; De Verdal et al., 2017; Zhang et al., 2017). Experts from the Animal Genetic Resources Committee of FAO also recommend RFI as an indicator for evaluating animal FE traits (Zhang, 2010).

The intestinal microbiota and the digestive enzyme activity in the intestines are regarded as two key factors in animal digestion (Motlagh et al., 2012; Fang et al., 2015). It is hypothesized that these two factors are strongly associated with the RFI of animals. However, there is an important symbiotic relationship between intestinal microbiota and hosts that contribute to energy balance, metabolism, intestinal health, immune activity, and neural development of hosts (Turnbaugh et al., 2007; Cho and Blaser, 2012; Shreiner et al., 2015). Intestinal microbiota are involved in the regulation of host energy-uptake efficiency, which was significantly associated with weight gain (Looft et al., 2014; Kim et al., 2015) and FE (Singh et al., 2012; Mccormack et al., 2017) and plays an important role in maintaining the stability of the intestinal environment (Chung and Kasper, 2010). Changes in intestinal microbiota could inevitably influence intestinal metabolic function and further affect the physiology of organisms (Levy et al., 2017), which can be partially indicated by intestinal metabolites (Yin et al., 2021). Therefore, numerous studies have analyzed intestinal microbiota structure and metabolites to explore the regulatory mechanisms of FE (Metzler-Zebeli et al., 2019; Gardiner et al., 2020).

Digestive enzyme activity also reflects the digestive and absorptive capacity of the intestine, which in turn determines feeding and growth of animals (Baiao et al., 2017; Ydsr et al., 2019). Digestive enzymes are known to influence diet, growth, and many other traits of abalone (Gao et al., 2016, 2021). In addition, intestinal microbiota can influence the production of some digestive enzymes to some extent (Ekhart et al., 2012).

Abalone is an economically important aquaculture mollusk with high-quality protein resources that is widely cultured around the world (Froehlich et al., 2018). Its feed cost has been elevated with the industrialized production mode and rising feed prices, constraining further development of the abalone aquaculture industry in China, whose abalone aquaculture production accounts for 90% of the world. Studies on the FE of abalone from the perspective of intestinal microbiota are limited, although other investigations have shown that intestinal microbiota are irreplaceable for the growth and immunity of abalone (Jing et al., 2012; Choi et al., 2021). In this study, intestinal microbiota, digestive enzyme activity, and metabolome of the Pacific abalone, *Haliotis discus hannai*, with high FE (low RFI) and low FE (high RFI) were compared, and their interconnections in the regulation of FE of abalone were explored.

## Materials and Methods

### Experimental Design and Sample Collection

A total of 648 Pacific abalone individuals were selected for the feeding experiment (average shell length: 5.1 cm, average wet weight: 17.8 g). Each abalone was fed in a separate breeding unit (including a breeding container and a breeding net bag). Each abalone was fed with an equal amount of enough dried kelp (approximately 7% of body weight) once every 3 days, and food debris was collected. The feed condition, water temperature (21 ± 1°C), and other factors were identical for all of the individuals. After 72 days of culture experiment, the average daily gain (ADG), daily feed intake (DFI), feed efficiency ratio (FER), and RFI of each abalone were calculated. FER is the ratio of ADG to DFI, while RFI is calculated by a multivariate non-linear model. Two groups of abalone (*N* = 8 individual/group) were selected according to their RFI values: group A [group high feed efficiency (HFE)] was defined as the high-FE group (The 8 abalone with the lowest RFI), and group B [group low feed efficiency (LFE)] was defined as the low-FE group (The 8 abalone with the highest RFI). The intestines of these abalone individuals were divided equally into two parts. One part was used for 16S rDNA amplicon sequencing, and other part was used for the determination of metabolome and enzyme activity after cleaning and scraping the contents (rinsed out with 0.84% iced saline). All of the intestine samples were flash frozen in liquid nitrogen and stored at −80°C before subsequent measurements.

### Intestinal Microbiome Analysis

Total microbe genome DNA of the intestine samples (*N* = 8 individual/group) from the two groups was extracted using the Cetyl trimethyl ammonium bromide/Sodium dodecyl sulphate (CTAB/SDS) method, and DNA concentration and purity were assessed on 1% agarose gels. Then, DNA was diluted to about 1 ng/μL using sterile water. The 16S rDNA genes were amplified using specific primers with the barcode (primer: 16S V3-V4: 341F-806R). All of the polymerase chain reaction (PCR) reactions were conducted in 30-μL volume, including 15 μL of Phusion^®^ High-Fidelity PCR Master Mix (New England Biolabs, Beijing, China), 0.2 μM forward and reverse primers, and about 10 ng template DNA. PCR products were mixed in equidensity ratios and purified, and the amplicon products were sequenced using an Illumina MiSeq platform.

Raw data was quality controlled (tags filtering and de-chimerise) by quantitative insights into microbial ecology (QIIME) (version 1.8.0) to obtain clean data. Sequence analysis was performed by using the UPARSE software package using the UPARSE-OTU and UPARSE-OUTref algorithms. In-house Perl scripts were used to analyze alpha and beta diversity. Sequences with ≥97% similarity were assigned to the same OTUs (Edgar, 2013). The unweighted unifrac distance for principal coordinate analysis (PCoA) was performed using the QIIME (version 1.8.0) software. Linear discriminant analysis effect size (LEfSe) analysis was performed using the *LEfSe* package in Python. This method was designed to provide biological class explanations to establish statistical significance, biological consistency, and effect-size estimation of the predicted biomarkers. Phylogenet icInvestigation of communities by reconstruction of unobserved states (PICRUSt) software was used to infer the functional gene composition of the samples by comparing the species composition information obtained from 16S sequencing data. Other statistical analyses were conducted in R (Segata et al., 2011).

### Untargeted Metabolite Profiling

Intestine samples (*N* = 8 individual/group) were homogenized with 200 μL H_2_O and five ceramic beads, followed by the addition of 800 μL methanol/acetonitrile (1:1, v/v). The mixture was centrifuged for 15 min (14,000*g*, 4°C), and the supernatant was dried in a vacuum centrifuge. The samples were re-dissolved in 100 μL acetonitrile/water (1:1, v/v) solvent for subsequent liquid chromatography mass spectrometry (LC-MS) analysis performed using an ultra high performance liquid chromatography (UHPLC) (1290 Infinity LC, Agilent Technologies, Shanghai, China) coupled to a quadrupole time-of-flight (AB Sciex TripleTOF 6600) in Shanghai Applied Protein Technology Co., Ltd., Shanghai. China.

ProteoWizard MSConvert, XCMS, and CAMERA software was used for discriminating and annotating metabolites based on an in-house database. After normalization to total peak intensity, the processed data were analyzed by the R *ropls* package, where it was subjected to orthogonal partial least-squares discriminant analysis (OPLS-DA). Seven-fold cross-validation and response permutation testing were conducted to evaluate the robustness of the model. The variable importance in the projection (VIP) value of each variable in the OPLS-DA model was calculated to determine its contribution to the classification. Metabolites with the VIP value >1 and *p*-value <0.05 were considered to be significantly different, and they were further searched against the kyoto encyclopedia of genes and genomes (KEGG) database for metabolic pathway analysis by the MetaboAnalyst 4.0 website.^1^ After that, potential biomarkers were identified using receiver operating characteristic (ROC) analysis by the R *pROC* package.

### Enzyme Activity

The activity of alginate lyase was measured using the 3,5-dinitrosalicylic acid (DNS) method (Bergmeyer, 1974). Briefly, 0.1 g tissue was mixed with 1 mL 0.1 mol/L phosphate buffer for homogenization, and it was centrifuged at 10,000*g* at 4°C for 10 min. The supernatant was reacted with 1% sodium alginate and 0.51% carboxymethyl cellulose for 30 min, and the absorbance at a wavelength of 520 nm was measured by a microplate reader. Pepsin, trypsin, cellulase, and α-amylase activities were determined using the corresponding kits (Nanjing Jiancheng Bioengineering Institute, Nanjing, China). Briefly, 0.2 g intestinal tissue per sample was homogenized with 1.8 mL 0.86% normal saline and then centrifuged at 2,500, 8,000, 8,000, and 2,500*g*/min for 15 min at 4°C to obtain the supernatant for subsequent analysis. One unit of pepsin activity is defined as 1 mg tissue broken down into 1 μg amino acids per min. One unit of trypsin activity is defined as 1 mg protein catalyzed with an increase in absorbance of 1 at a wavelength of 253 nm. One unit of cellulase activity is defined as per g tissue to catalyze 1 μg glucose per min. One unit of α-amylase activity is defined as 1 mg reducing sugar catalyzed per min per unit of tissue (Gao et al., 2021). Protein content in the homogenate was determined using Coomassie blue staining, with bovine serum albumin as the protein marker (Bradford, 1976).

### Statistical Analysis

Differences in traits (RFI, ADG, FER, and DFI) and enzyme activity between the high- and low-FE groups were analyzed using independent sample *t*-tests completed by the statistical product and service solutions (SPSS) software. The correlations between significantly different microbiome and significantly different metabolites screened in the experimental samples were indicated by Spearman’s rank correlation coefficient. Hierarchical clustering analysis was conducted in R and Cytoscape software, respectively.

## Results

### Feed Efficiency Phenotype

The traits associated with FE were calculated for both group A and group B (Table 1). The RFI values of group A were significantly higher than these of group B (*P* < 0.05). Although the mean ADG value of group A was 1.4 times higher than that of group B, the difference was not significant (*P* > 0.05). There was a weak difference in DFI values between the two groups.

**TABLE 1:** Phenotype statistics of two groups of abalone after a 72-day culture experiment.

	Group A (*N* = 8)			Group B (*N* = 8)		
	Mean	Max	Min	Mean	Max	Min
FER	0.427^a^	0.610	0.157	0.296^ab^	0.424	0.020
RFI (g/day)	−0.029^a^	−0.026	−0.378	0.022^b^	0.032	−0.005
ADG (g/day)	0.200^a^	0.255	0.071	0.143^ab^	0.212	0.011
DFI (g/day)	0.467^a^	0.490	0.443	0.488^ab^	0.530	0.474

*RFI, residual feed intake; FER, feed efficiency ratio; ADG, average daily gain; DFI, daily feed intake; A group, group with low RFI; B group, group with high RFI; a,b, lowercase letters in the same row indicate differences between the two groups (P < 0.05).*

### Changes in the Intestinal Microbiome of Abalone With Different Feed Efficiency

**FIGURE 1:**
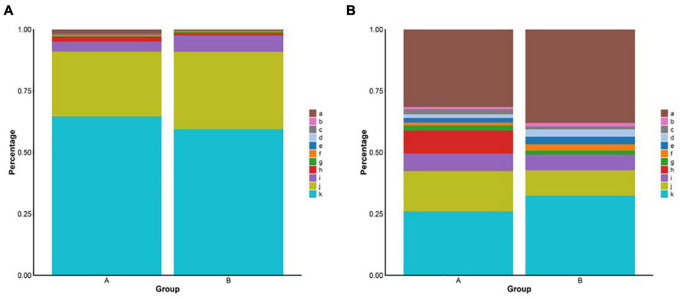
**(A)** Relative abundance of bacteria phyla from the group A and group B. a: Others, b: *Deinococcus-Thermus*, c: *Patescibacteria*, d: *Epsilonbacteraeota*, e: *Spirochaetes*, f: *Actinobacteria*, g: *Cyanobacteria*, h: *Firmicutes*, i: *Bacteroidetes*, j: *Tenericutes*, and k: *Proteobacteria.* **(B)** Relative abundance of bacteria genus from different groups. a: Others, b: *Formosa*, c: *Caulobacter*, d: *Ruegeria*, e: *Halocynthiibacter*, f: *Tamlana*, g: *Pseudoalteromonas*, h: *Cobetia, i: uncultured*, j: *Aquabacterium*, and k: *Mycoplasma.*

The coverage indices of the two groups were >99% (Table 2). Based on the 97% sequence similarity, the number of operational taxonomic unit (OTU) samples ranged from 1,367 to 1,749 (Supplementary Figure 1A). Group A was higher than group B in microbiota diversity (Shannon), microbiota richness (Ace and Chao1), and phylogenetic diversity (PD whole tree) (Table 2). *Proteobacteria, Tenericutes*, and *Bacteroidetes* were the three predominant microbes identified in the two groups at the phylum level, accounting for more than 95% of the sequences. However, the proportion of predominant microbes in the two groups was different from the proportion of *Proteobacteria* in group A (64.6%), which was higher than that in group B (59.4%). The proportion of *Tenericutes* in group B (31.5%) was higher than that in group A (26.3%) (Figure 1A). The intestinal microbiota was predominated by *Mycoplasma*, *Aquabacterium*, *Cobetia*, and *Pseudoalteromonas* at the genus level. In addition, the percentages of *Pseudoalteromonas* and *Cobetia* were higher in group A than in group B (Figure 1B). The proportions of the different intestinal microbes varied between individuals, but the predominant microbes were almost consistent (Supplementary Figures 1C,D).

**TABLE 2:** α-Diversity indexes of the two study groups.

Groups	Coverage	Shannon	Simpson	Ace	Chao1	PD whole tree
A	0.998	4.1	0.84	603.9	604.3	101.2
B	0.998	3.97	0.86	517.7	517.9	54.0

**FIGURE 2:**
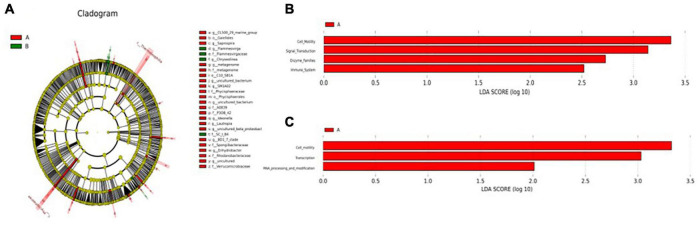
**(A)** Cladogram showing significant overrepresentation of microbial populations and taxa as assessed by linear discriminant analysis effect size (LEfSe) (*P* ≤ 0.05 and linear discriminant analysis (LDA) cutoff >3.0). **(B)** Microbial functional predictions revealed the most differentially regulated metabolic pathways in the fecal microbiome at KEGG level. **(C)** Microbial functional predictions revealed the most differentially regulated metabolic pathways in the fecal microbiome at the clusters of orthologous groups (COG) level.

Principal coordinate analysis (PCoA) analysis was conducted to further evaluate the dissimilarity of bacterial composition among the two groups. Significant difference (*P* < 0.05) in beta diversity between the two groups. Although individuals in group A were more dispersed, individuals in group B could be well clustered together (Supplementary Figure 1B). To identify specific microbiota characteristics of the two groups, linear discriminant analysis (LDA) analysis coupled with LEfSe was performed, which identified 24 (A) and 4 (B) biomarkers that enriched the two groups, which mainly included *Saprospira*, *Chryseolinea*, *uncultured_bacterium*, *Ideonella*, and *Rhodanobacteraceae* (Figure 2A). These biomarkers in the two groups were enriched in cell motility, signal transduction, enzyme families, and immune system according to the KEGG function prediction (Figure 2B) and enriched in cell motility, transcription, and RNA processing, and modification according to the clusters of orthologous groups (COG) function prediction (Figure 2C).

### Changes in the Intestinal Metabolites of Abalone With Different Feed Efficiency

To identify metabolites that might play roles in intestinal homeostasis, a non-targeted metabolomics analysis based on an UHPLC-quadrupole time-of-flight tandem mass spetrometry (QTOF-MS/MS) platform and self-compiled metabolite database was conducted in both positive and negative ion modes. A total of 384 metabolites were detected (Supplementary Figure 2), which mainly included carboxylic acids and derivatives (159), organooxygen compounds (48), fatty acyls (33), organonitrogen compounds (17), and purine nucleosides (9). The peaks extracted from all of the experimental samples and quality control (QC) samples were analyzed by principal component analysis (PCA), and the QC samples were clustered together and located between the experimental groups, indicating good reproducibility of the experiments. In addition, the identification level of the resulting metabolites was above Level 2.

**FIGURE 3:**
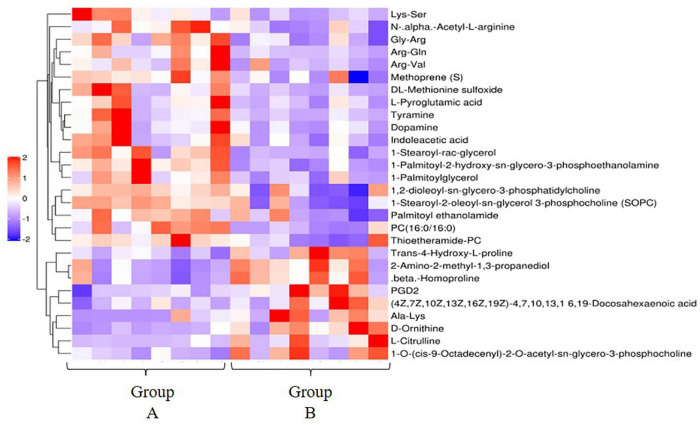
Hierarchical clustering analysis of the differential metabolites in the high- and low-residual feed intake (RFI) groups in the positive ion mode. Red and blue respectively represent upregulated and downregulated differential metabolites (DEs).

**FIGURE 4:**
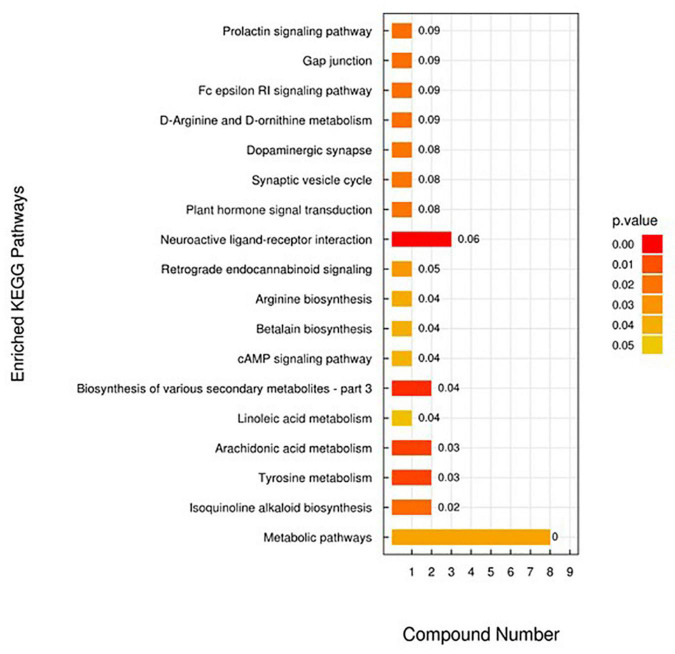
Upregulated and downregulated metabolic pathways in abalone from groups A and B.

The results of OPLS-DA analysis can well explain and predict differences (both positive and negative ion mode) between the two groups (Supplementary Figure 3). A total of 28 differentially expressed metabolites (DEMs) in the positive ion mode (19 significantly upregulated and 9 significantly downregulated) (Figure 3) and 1 DEM in the negative ion mode (1 significantly downregulated) were identified. DEMs in the positive ion mode enriched 18 KEGG pathways, including metabolic pathways [PC (16:0/16:0), 1-stearoyl-2-oleoyl-sn-glycerol 3-phosphocholine, L-citrulline, tyramine, D-ornithine, prostaglandin D (PGD2), indoleacetic acid, L-pyroglutamic acid, and dopamine], tyrosine metabolism (tyramine and dopamine), and arachidonic acid metabolism [PC (16:0/16:0), 1-stearoyl-2-oleoyl-sn-glycerol 3-phosphocholine (SOPC), and PGD2] (Figure 4).

Receiver operating characteristic (ROC) curves were generated to assess the potential usefulness of metabolites as biomarkers for high and low FE. A total of 17 metabolites (AUC > 0.9) were screened in the comparison of group A and group B. Of them, 1-palmitoyl-2-hydroxy-sn-glycero-3-phosphoethanolamine, 3,3-dimethylacrylic acid, Phe-Gly, and indoxyl sulfate have the optimal classification effect (AUC > 0.95). Secondly, Arg-Gln, PC (16:0/16:0) also have a good classification effect (AUC > 0.90) (Supplementary Material).

### Relationship Between Intestinal Microbiome and Metabolites

**FIGURE 5:**
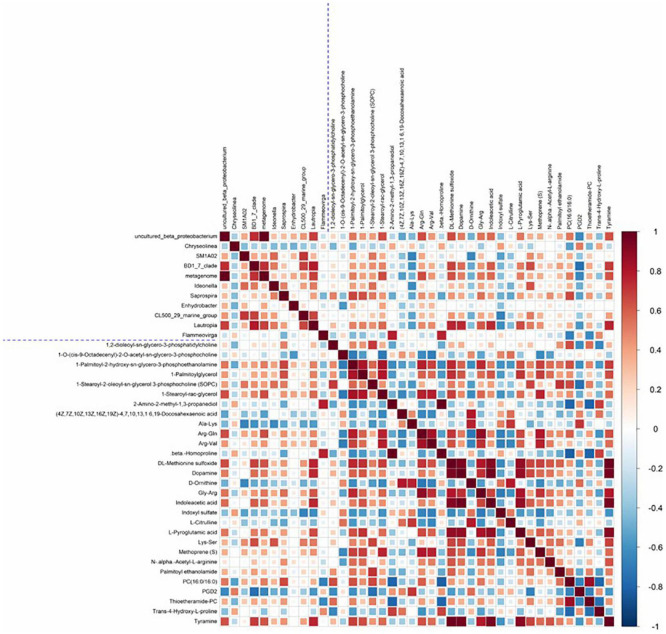
Heat map of the Spearman correlation coefficient matrix for significantly different intestinal microbiota and significantly different metabolites. All intestinal microbiota and metabolites shown in Figure were significantly different between the two groups. The blue dashed line in the middle is the dividing line, and the correlation coefficient matrix heat map can be divided into four quadrants, with the upper left corner showing correlations between different intestinal microbiota, the lower right corner showing correlations between significantly different metabolites, and both the upper right and lower left corners showing correlations of significantly different intestinal microbiota and significantly different metabolites, with mirror symmetry.

In the comparison between groups A and B, a hierarchical clustering heat map of significantly different intestinal microbiota and significantly different metabolites is shown in Figure 5. A total of 71 pairs of significantly related differential microbiota and metabolites were found. Intestinal microbiota *uncultured beta proteobacterium* showed a significant positive correlation with the metabolites DL-methionine sulfoxide, Arg-Gln, L-pyroglutamic acid, dopamine, tyramine, PC (16:0/16:0), and indoleacetic acid (*r* > 0.5, *P* < 0.05). Intestinal microbiota *BD1_7_clade* showed a significant positive correlation with the metabolites DL-methionine sulfoxide, dopamine, indoleacetic acid, Lys-Ser, and tyramine. In addition, there was a high correlation between *Lautropia* and the intestinal metabolites DL-methionine sulfoxide, dopamine, indoleacetic acid, L-pyroglutamic acid, and tyramine (*r* > 0.6, *P* < 0.05).

### Digestive Enzyme Activities

**FIGURE 6:**
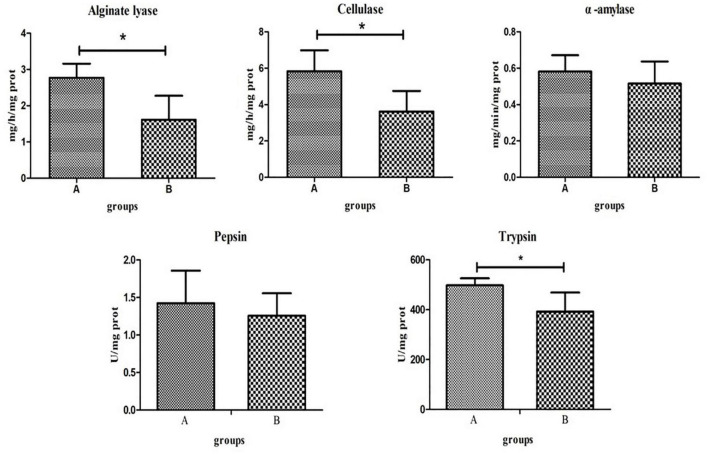
Key digestive enzyme activity and protease activity in the intestines of the high- and low-residual feed intake (RFI) groups. “*” represents a significant difference (*P* < 0.05).

Digestive enzyme activities of the two groups significantly differed. *T*-tests showed that the activities of alginate lyase, cellulase, and trypsin in group A were significantly higher than those in group B (*P* < 0.05). The activities of α-amylase and pepsin were higher in group A than in group B, although this was not statistically significant (*P* > 0.05; Figure 6).

## Discussion

In this study, the hypothesis that microbiota structure, digestive enzyme activity, and metabolome in the intestines are associated with FE of animals was tested. For the assessment of growth and resistance traits in abalone, the feeding experiment period was generally 60–90 days (Huang et al., 2018; Wang et al., 2020). In this experiment, a 72-day feeding experiment period was sufficient to obtain growth and feeding efficiency traits for abalone. Low-RFI abalone individuals were selected as the high-FE group (group A) and high-RFI abalone individuals were selected as the low-FE group (group B). The RFI values of group B were significantly higher than those of group A; however, there was no significant difference between the two groups in ADG, FER, and DFI, possibly because of the small number of individuals. It has been reported that FER is correlated to ADG (Case et al., 2012), whereas RFI has a weak correlation to ADG (Dai et al., 2017). Each abalone in the experiment was raised individually, with adequate food and no competition, which may account for the small difference in DFI.

Recently, the development of high-throughput sequencing has facilitated comprehensive profiling of the microbiota co-occurring in the intestines of the animal (Argumedo-Hernández et al., 2010; Zhao et al., 2018). Diet, living environment, and other factors greatly impact the animal intestinal microbiota (Looft et al., 2014; Singh et al., 2014; Kim and Isaacson, 2015). This is one of the first studies to exploit this technology to examine the intestinal microbiota in abalone with varying FEs. The two groups of abalone in this experiment were subjected to the same management, environmental, and nutritional conditions to eliminate additional effects. The intestinal microbiota of all of the abalones were predominated by the phyla *Proteobacteria* and *Tenericutes*, which is consistent with previous findings (Wang et al., 2020; Choi et al., 2021). *Proteobacteria* are carbohydrate-fermenting bacteria and a high percentage in the intestines of abalone enhances the ability to utilize carbohydrates and improve production performance (Elolimy et al., 2019). Abalones with higher FE had a higher percentage of *Proteobacteria*, which is consistent with the results in the study on the high-feed conversion ratio (FCR) and low-FCR broiler growers (Singh et al., 2012). In addition, changes in the abundance of *Firmicutes* may affect energy harvesting ability (Turnbaugh, 2006), which may account for the high abundance of *Firmicutes* in the high-FE group.

At the genus level, *Mycoplasma* is the most abundant bacteria and has been reported several times in abalone (Cicala et al., 2018; Nam et al., 2018). *Pseudoalteromonas* and *Cobetia* accounted for a higher proportion of the intestinal microbiota in group A than in group B, and both groups could produce alginate lyase (Chaki et al., 2008; Gong et al., 2016). Alginate lyase, a digestive enzyme catalyzing the degradation of alginate, has been isolated from various kinds of organisms with different substrate specificities and is extremely important in food digestion and nutrition absorption in abalone (Zhu and Yin, 2015; Bansemer et al., 2016). The higher proportion of *Pseudoalteromonas* and *Cobetia* in the high-FE group may have increased the activity of alginate lyase in abalone, which promoted the digestion and absorption of food and thus improved FE. The presumed possibility was initially established by measuring intestinal enzyme activity, although the gene for alginate lyase is present in abalone (Sim et al., 2012). Combined with the results of other digestive enzyme activities and protein enzyme activities, we hypothesized that alginate lyase and cellulase enzyme activities may play important roles in inducing changes in RFI in abalone. However, whether the increase in cellulase enzyme activity was influenced by the intestinal microbiota needs to be further investigated.

Microbial culture analysis of healthy and diseased abalone intestines revealed a decrease in microbiota diversity in diseased abalone (Huang et al., 2010) and we theorize that microbiota diversity may represent the health condition of the abalone intestine. The reduction in intestinal microbiota diversity in group B may be an indication of abnormal intestinal health conditions, which in turn contributes to the low-FE trait in abalone. Another study showed that the addition of probiotics to feed promoted growth, immunity, and intestinal microbial richness in the small abalone *Hediste diversicolor* (Zhao et al., 2018), again suggesting that high microbiota diversity is associated with positive aspects of the trait. Abalone individuals with high FE had high microbiota diversity and richness, which is consistent with the results in most animal studies (Bergamaschi et al., 2020).

By establishing associations between microbiota and the FE of abalone, a number of microbiota biomarkers were found in the high- and low-FE groups, which could provide a reference for screening abalone with high FE and improve the FE traits of abalone through targeted regulation of intestinal microbiota. The biomarker *Saprospira* between groups A and B is a member of *Bacteroidetes*, and members of this bacteria are closely associated with intestinal microbiota in pigs with low RFI (Mccormack et al., 2017), indicating the strong ability of such bacteria to ferment complex carbohydrates (UmuJeremy et al., 2015). *Rhodanobacteraceae* are considered to be potentially beneficial bacteria because of their ability to produce beneficial chemicals (Saudo-Wilhelmy et al., 2013). *Rhodobacteraceae* has the role of catalytic denitrification, degradation of various organic pollutants, and inhibition of the growth of harmful pathogens (Liu et al., 2012; Wang et al., 2018). Therefore, we hypothesized that the immunity of abalone in group A that was enriched with *Rhodobacteraceae* was higher than that of group B, which in turn influenced FE, and this hypothesis coincided with the results of upregulation of the immune pathway in group A. *Actinobacteria* has been recognized as an important source of biologically active natural products with a variety of biological properties such as antibacterial, anticancer, and antiviral (Manivasagan et al., 2014). The biomarkers *Llumatobacteraceae* and *Gaiellales* in group A belong to *Actinobacteria*. Taken together, we hypothesized that *Saprospira*, *Rhodanobacteraceae*, *Llumatobacteraceae*, and *Gaiellales* in the intestines may potentially be utilized as biomarkers for distinguishing high-FE abalone from low-FE abalone. Improvement of intestinal microbiota through the transplantation of specific bacteria or the use of dietary supplements has progressed in other species (Vos, 2013; Wlodarska et al., 2015). Therefore, it is possible to improve the FE of abalone by regulating the intestinal microbiota composition.

Metabolite identification is the main metabolomic bottleneck, especially in marine non-model organisms (Reverter et al., 2020). In this study, the intestinal metabolome of abalone in the high- and low-FE groups was analyzed, and a total of 344 metabolites were identified. The number of metabolites identified was similar to previous studies on abalone (Shen et al., 2021), and major metabolites were identified, including taurine, citrate, succinate, lactate, glutamate, glutamine, and L-carnitine, thus confirming the reliability of our results. Arginine is involved in RNA synthesis and protein glycosylation, and it is an essential amino acid for cellular function (Li et al., 2018). The growth and FE of red drum (*Sciaenops ocellatus*) have a standard requirement for arginine (Barziza et al., 2000). In this study, the arginine synthesis pathway was upregulated in the low-RFI group, and we hypothesized that arginine plays a regulatory function in abalone FE. Furthermore, differences in amino acid and fatty acid metabolic pathways indicate the importance of intestinal metabolic capacity on FE traits in abalone. This study was also the first to elucidate the mechanisms influencing feed efficiency traits in abalone through metabolome profiles.

Changes in intestinal microbiota may depend on or in turn influence metabolite production in the host (Levy et al., 2017). Therefore, changes in metabolites may be closely related to the intestinal microbiota. *Proteobacteria* in the intestines has been reported to play a crucial role in amino acid utilization (Neis et al., 2015). The three intestinal bacteria (*Uncultured beta proteobacterium*, *BD1_7_clade*, and *Lautropia*) that were significantly different in the high-/low-FE groups all belonged to *Proteobacteria*. DL-methionine sulfoxide is involved in the methionine metabolic pathway, which promotes protein synthesis in animal growth (Hu et al., 2016) and production performance (Fagundes et al., 2016). In addition, methionine is required for the biosynthesis of spermidine and spermine, two compounds that help to reduce oxidative stress (Minois et al., 2011). Indoleacetic acid, a metabolite of tryptophan, is a ligand of the aromatic hydrocarbon receptor (AHR) (Roager and Licht, 2018), a transcription factor that is widely expressed in cells of the immune system. Several studies have shown that AHR activation alters innate and adaptive immune responses in a ligand-specific manner. In addition, another important physiological function of tryptophan is protein synthesis (Gargaro et al., 2016). L-pyroglutamic acid is a metabolite in the glutathione cycle, which can be converted to glutamate by the 5-oxoprolinase enzyme (Mello et al., 1995). The addition of glutamate in feed can increase the FE of animals (Cantalapiedra-Hijar et al., 2016). The tyrosine metabolic pathway is upregulated in pigs with low RFI (Mccormack et al., 2017), in which tyrosine and its subsequent metabolites can reduce the production of reactive oxygen species (ROS), and the upregulation of tyrosine metabolism may play a positive role in reducing oxidative damages in the intestines (Beloborodova et al., 2012). This provides evidence that the intestines or microbiota of abalones that are more feed efficient could potentially be “healthier.” Tyramine (TA), as a neurotransmitter, neuromodulator, and neurohormone, controls or regulates many behavioral and physiological processes in animals (Orchard, 1982). The nerve tissues of invertebrates, such as insects, crustaceans, and mollusks, contain large amounts of TA (Robertson and Juorio, 1976). Tyrosine is also required for the synthesis of 5-hydroxytryptamine and dopamine, both of which are important neurotransmitters (Bergwerff et al., 2016). The upregulation of tyrosine and tryptophan in the HFE group may be an adaptation, which promotes metabolism in organisms. DL-methionine, indoleacetic acid, and L-pyroglutamic acid were positively correlated with *Proteobacteria* (*Uncultured beta proteobacterium*, *BD1_7_clade*, and *Lautropia*) (Figure 5), and it was hypothesized that the above three intestinal bacteria contributed to the FE of abalone through the following two reasons. Firstly, *Proteobacteria* promote the utilization of amino acids in abalone, which in turn provides more nutrients to maintain the synthesis of amino acids required for abalone growth, ultimately improving the feed efficiency traits. Of course, the increased protease activity of the HFE group also promoted amino acid hydrolysis and protein absorption (Kumlu and Jones, 2010). Second, *Proteobacteria* improves the immunity of the abalone and reduces oxidative damage of the intestine to ensure that the intestine performs its digestive and absorption functions better, which in turn improves the feed efficiency of the abalone. In summary, we hypothesized that the microbiota, metabolism, and enzyme activity in the abalone intestines interact to alter amino acid metabolism, immune responses, and signaling pathways, which in turn influence FE.

## Conclusion

This study reveals for the first time differences in intestinal microbiota structures and intestinal metabolism in shellfish with different feed efficiencies. Abalone with high FE have higher microbiota diversity and a higher proportion of *Proteobacteria* and *Firmicutes*. Four microbiota, namely, *Saprospira*, *Rhodanobacteraceae*, *Llumatobacteraceae*, and *Gaiellales*, may be potentially utilized as biomarkers for distinguishing between high- and low-FE abalone. The intestinal digestive enzyme activity also contributed to differences in FE in abalone, and *Pseudoalteromonas* and *Cobetia* may influence the activity of alginate lyase in the intestine. The interaction between microbiota and intestines influences amino acid metabolism, immune response, and the signal transduction pathway in abalone, in turn causing differences in FE. The differences in amino acid metabolism may also be influenced by intestinal trypsin activity. This study not only analyzed the mechanisms of variations in abalone FE in terms of intestinal microbiota, intestinal metabolism, and intestinal enzyme activity, but it also provided important basic knowledge for improving abalone FE by modulating intestinal microbiota.

## Data Availability Statement

The data presented in the study are deposited in the GenBank Sequence Read Archive (SRA) repository, accession number PRJNA805375.

## Ethics Statement

The animal study was reviewed and approved by the ethics committee of Xiamen University.

## Author Contributions

CK, WwY, and WcY provided the experimental ideas and design of this study. WcY, MZ, JL, FY, ZH, and WZ did the experiments. YL and SG helped to analyze the data. WcY and YL wrote the manuscript. WwY, YS, and XL revised the manuscript. All authors approved the final manuscript.

## Conflict of Interest

The authors declare that the research was conducted in the absence of any commercial or financial relationships that could be construed as a potential conflict of interest.

## Publisher’s Note

All claims expressed in this article are solely those of the authors and do not necessarily represent those of their affiliated organizations, or those of the publisher, the editors and the reviewers. Any product that may be evaluated in this article, or claim that may be made by its manufacturer, is not guaranteed or endorsed by the publisher.

## Footnotes

1
https://www.metaboanalyst.ca/
