# Supplementary material for: Exploring the Intestinal Microbiota and Metabolome Profiles Associated With Feed Efficiency in Pacific Abalone (Haliotis discus hannai)
Source: Front Microbiol. 2022 Mar 17;13:852460. doi: 10.3389/fmicb.2022.852460 (PMC8969561; doi:10.3389/fmicb.2022.852460)
Supplement: Supplementary file 2 [file Data_Sheet_2.docx]

Figure


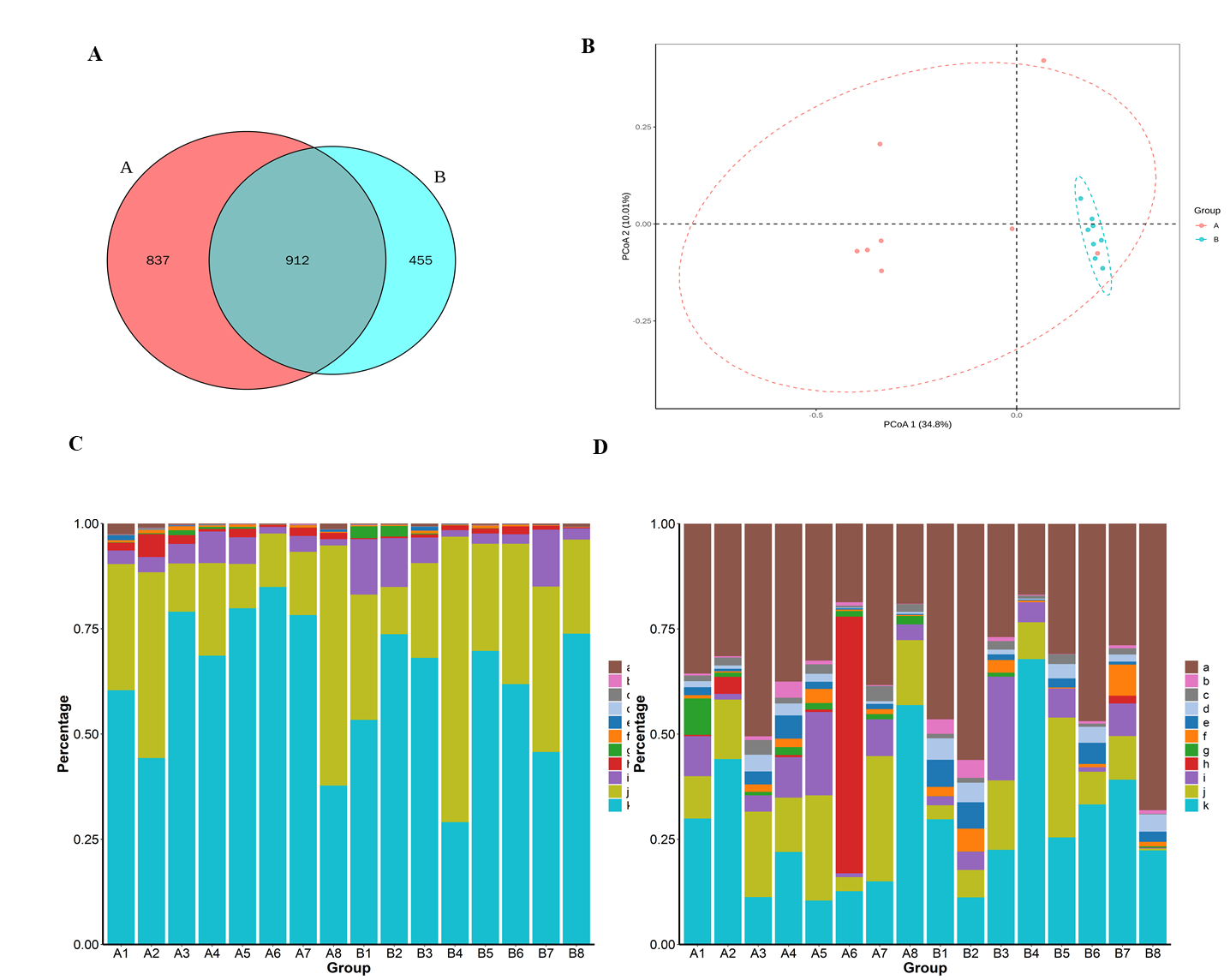


**Figure S1.** (A) Clustering analysis of OTUs for high- and low-RFI groups. (B) Principal coordinate analysis (PCoA) plot (based on OTUs) for high- (n = 8) and low-RFI (n = 8) groups. (C) Relative abundance of bacteria phyla for all samples. a: Others, b: *Deinococcus-Thermus*, c: *Patescibacteria*, d: *Epsilonbacteraeota*, e: *Spirochaetes*, f: *Actinobacteria*, g: *Cyanobacteria*, h: *Firmicutes*, i: *Bacteroidetes*, j: *Tenericutes*, k: *Proteobacteria.* (D) Relative abundance of bacteria genus for all samples. a: Others, b: *Formosa*, c: *Caulobacter*, d: *Ruegeria*, e: *Halocynthiibacter*, f: *Tamlana*, g: *Pseudoalteromonas*, h: *Cobetia, i: uncultured*, j: *Aquabacterium*, k: *Mycoplasma.*


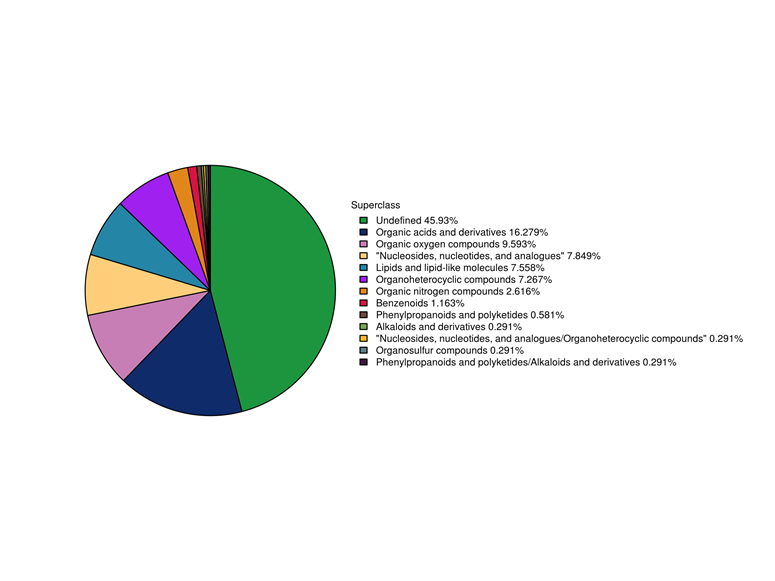


**Figure S2**. Proportion of identified metabolites in each chemical classification.


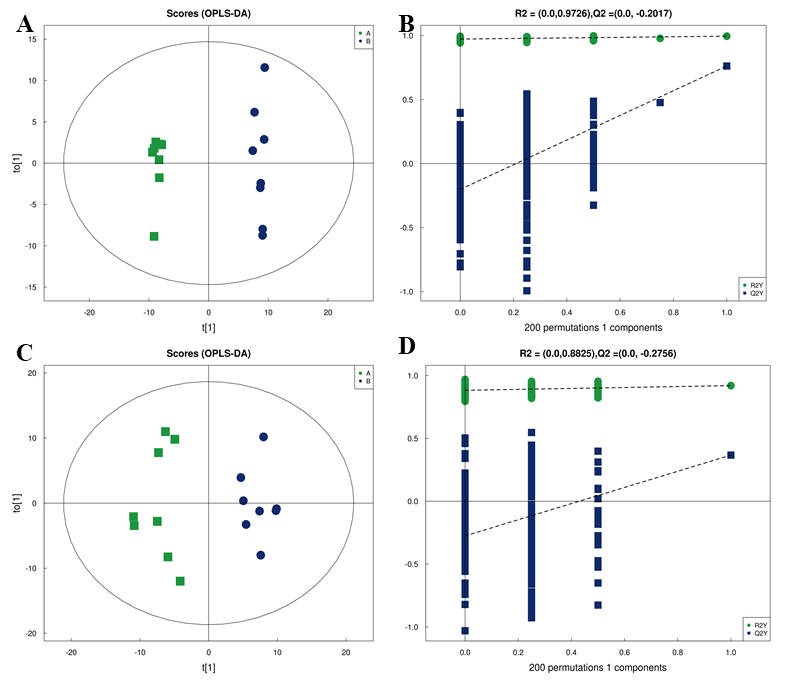


**Figure S3.** (A) OPLS-DA in positive ion mode. (B) Positive ion mode OPLS-DA displacement test. (C) OPLS-DA in negative ion mode. (D) Negative ion mode OPLS-DA displacement test.
